# Cerebral Activations Related to Ballistic, Stepwise Interrupted and Gradually Modulated Movements in Parkinson Patients

**DOI:** 10.1371/journal.pone.0041042

**Published:** 2012-07-23

**Authors:** Carolien M. Toxopeus, Natasha M. Maurits, Gopal Valsan, Bernard A. Conway, Klaus L. Leenders, Bauke M. de Jong

**Affiliations:** 1 Department of Neurology, University Medical Center Groningen, University of Groningen, Groningen, The Netherlands; 2 Bioengineering Unit, University of Strathclyde, Glasgow, United Kingdom; The University of Western Ontario, Canada

## Abstract

Patients with Parkinson’s disease (PD) experience impaired initiation and inhibition of movements such as difficulty to start/stop walking. At single-joint level this is accompanied by reduced inhibition of antagonist muscle activity. While normal basal ganglia (BG) contributions to motor control include selecting appropriate muscles by inhibiting others, it is unclear how PD-related changes in BG function cause impaired movement initiation and inhibition at single-joint level. To further elucidate these changes we studied 4 right-hand movement tasks with fMRI, by dissociating activations related to abrupt movement initiation, inhibition and gradual movement modulation. Initiation and inhibition were inferred from ballistic and stepwise interrupted movement, respectively, while smooth wrist circumduction enabled the assessment of gradually modulated movement. Task-related activations were compared between PD patients (N = 12) and healthy subjects (N = 18). In healthy subjects, movement initiation was characterized by antero-ventral striatum, substantia nigra (SN) and premotor activations while inhibition was dominated by subthalamic nucleus (STN) and pallidal activations, in line with the known role of these areas in simple movement. Gradual movement mainly involved antero-dorsal putamen and pallidum. Compared to healthy subjects, patients showed reduced striatal/SN and increased pallidal activation for initiation, whereas for inhibition STN activation was reduced and striatal-thalamo-cortical activation increased. For gradual movement patients showed reduced pallidal and increased thalamo-cortical activation. We conclude that PD-related changes during movement initiation fit the (rather static) model of alterations in direct and indirect BG pathways. Reduced STN activation and regional cortical *increased* activation in PD during inhibition and gradual movement modulation are better explained by a dynamic model that also takes into account enhanced responsiveness to external stimuli in this disease and the effects of hyper-fluctuating cortical inputs to the striatum and STN in particular.

## Introduction

Successful motor performance in daily life implies that movements are adequately tuned to external conditions, particularly experienced by visual cues [1]–[3]. Such performance requires a well-proportioned balance between initiation and inhibition of movement. In abrupt starting and stopping of movement, simultaneous contraction and relaxation of opposed muscle groups is obvious. Smooth movement execution, on the other hand, is achieved by gradual modulation of agonist and antagonist muscle activities. In task-related motor control, one may thus distinguish two levels of ongoing movement adjustments: (i) overall visuomotor control and (ii) co-ordination between various muscle groups to achieve a distinct movement. Regarding the latter, an adequate balance between initiation and inhibition is required to achieve the purpose of the intended movement [4]–[8]. For example, a fast reach to catch a dropped object mainly involves abrupt initiation of agonist activation, whereas gentle object manipulation or smooth handwriting requires gradual agonist-antagonist adjustment achieved by the timed selection of specific muscles [5], [9], [10].

At simple movement level, the basal ganglia (BG) and interconnected circuitry play a key role in the selection of appropriate muscles and inhibition of undesired motor activity [11]–[16]. The role of the BG in tasks constituted by more complex movement patterns entails continuous modification of smooth movement, requiring a gradual selection of assemblies of muscle synergies. The precise function of the BG in the organization of initiation and inhibition of movement, however, is not clear and is quite complex [9], [17]–[20].

The prominent contribution of the BG to the organization of movement initiation and inhibition is also revealed by BG dysfunction evidenced by the symptoms and movement impairments in Parkinson’s disease (PD) [21]–[24]. Degeneration of the brain stem substantia nigra causes striatal dysfunction in PD [25] with impairment of movement initiation and inhibition as classical features. These impairments can be task-related, e.g. difficulty to start/stop walking [26], whereas at single-joint level, impaired movement initiation [27] is also associated with insufficient inhibition of the antagonist muscle [28]. This suggests a relation with the clinical presentation of rigidity. Similarly, decreased ability of PD patients to perform movements smoothly [29] points at impaired gradual modulation of movement [30]. Dysfunction of BG input nuclei (striatum) leads to enhanced inhibition by BG output nuclei and subsequently reduced cortical activation [22], [31]. Although this ‘classical’ model may explain impaired movement initiation in PD, it does not fully explain insufficient movement inhibition and the poor gradual modulation of muscle synergies during movement execution in these patients [32].

The present functional magnetic resonance imaging (fMRI) study primarily aimed to identify PD-related changes in BG function involved in initiation, inhibition and gradual modulation of opposed muscle activity. To that end, we employed four manual movement tasks, characterized by abrupt starting and stopping during simple movements or compound muscle activities during more complex movements. All movement patterns concerned the same joint (the right wrist). Two of the present tasks were conceptually similar to tasks that we previously used to identify BG activations related to abrupt movement initiation and inhibition in healthy subjects [9]. In this respect, movement inhibition in our stop task concerned termination of ongoing movement and not the suppression of unwanted movement initiation. A novelty of the present study was the use of a manipulandum, with movement registration that also enabled visual feedback to the subject. In addition to abrupt flexion-extension tasks with movements along a single axis, we included two tasks requiring more elaborate adjustments in muscle activities. A task consisting of continuous circle movement was characterized by gradual modulation of muscle activity since it had no abrupt transitions [30]. However, this task also required more visuomotor control than the simple flexion-extension tasks, which implied that both gradual movement modulation and visuomotor transformation characterized this task as more complex than the flexion-extension tasks. A fourth experimental task (multi-directional, point to point step-tracking) was similarly associated with a high level of visuomotor complexity. Alike the circle task, multi-directional step-tracking movements result from variable muscle synergies needed to move the manipulandum towards different cued positions. However, in contrast to circle movement, this step-tracking task [33] included abrupt initiation and termination of movement. Specific comparisons between the experimental tasks (using a block-design analysis) enabled dissociation of BG activations related to modulation of compound muscle activities underlying movement execution from those related to the enhanced demand of visuomotor transformations.

Aside from the role of the BG in movement selection as discussed above, particularly the striatum may contribute to facilitation of cortico-cortical interactions required for visuomotor integration [14], [34]. The role of the striatum in visuomotor control is further revealed in PD patients who are more dependent on visual cues during movement execution [35]–[39]. This implies that a dissociation of activations in BG and interconnected cortical circuitry related to the two levels of motor organization, i.e. simple flexion-extension movements versus movements requiring more visuomotor control, may provide more insight in both the impairment of simple movement and altered visuomotor control in PD patients. Thus, by using tasks with common general characteristics, carried out along the same joint, the present study allowed to disentangle (i) BG activations related to basic movement selection from (ii) activations related to higher order motor control implicated in visuomotor transformations. With this approach we expected to further elucidate altered organization of movement initiation/inhibition in Parkinson’s disease.

## Materials and Methods

### Ethics Statement

The study was approved by the Medical Ethical Committee of the University Medical Center Groningen. Both healthy subjects and PD patients gave written informed consent in accordance with the Declaration of Helsinki (2008) prior to participation. All patients provided written informed consent.

### Subjects

Thirteen patients with idiopathic PD experiencing mild to moderate clinical symptoms were recruited. Patients were assessed by the Unified Parkinson’s Disease Rating Scale (UPDRS) [40] and Hoehn and Yahr disability scale [41]. In addition, nineteen healthy age and gender- matched subjects were recruited for participation. Patients had to be stable and had to refrain from taking their morning dose of levodopa, or dopamine agonists (overnight withdrawal) in order to reduce medication effects. Subjects had to be right handed as assessed by the Annett Handedness Scale [42]. Exclusion criteria for both groups were a history of epileptic seizures, head injury, neurological diseases (for patients: other than PD), psychiatric diseases or the use of medication affecting the central nervous system. Subjects with Mini Mental State Examination (MMSE [43]) scores below 25 were excluded. Patients who could not abstain from their levodopa use were excluded. Additionally, patients with Parkinsonism other than PD, or the tremor-dominant type of PD were excluded from participation in the study to obtain a maximally homogeneous group of patients; tremor-dominant PD might be regarded as a PD subtype [43]. All subjects came for two visits on separate days with a maximum interval of two weeks. During the first visit subjects were screened neurologically (performed by CMT) and practised the task.

### Experimental Design

**Figure 1:**
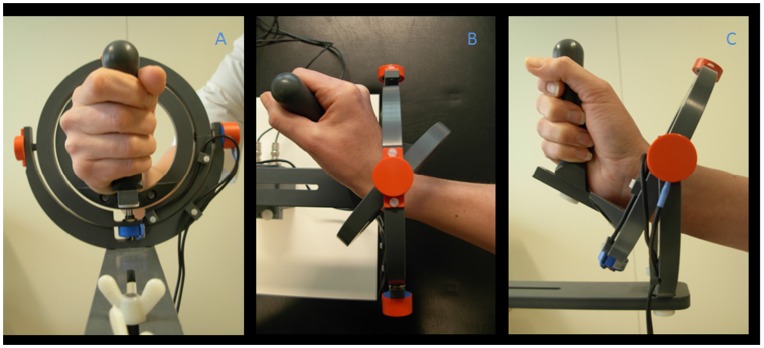
Photograph of the wrist manipulandum. The manipulandum consists of two concentric rings moving around perpendicular axes and allowing two degrees of freedom for wrist movement: wrist flexion-extension, ulnar-radial deviation and all combinations thereof. a: (frontal view) neutral position (origin), right hand positioned in a vertical plane holding the grip of the manipulandum; b: (top view) full wrist extension and c: (side view) full radial deviation.

All subjects performed four different movement tasks with the right hand using a magnetic resonance (MR) compatible manipulandum, in function similar to the manipulandum described by Hoffman and Strick for their studies on step-tracking [33] (fig. 1). The manipulandum consisted of a joystick-like device that enabled movements in two perpendicular planes allowing wrist flexion-extension, wrist ulnar-radial deviation and all combinations thereof. The right wrist joint was positioned in the center of the two concentric rings of the device, while the fingers were holding the grip of the manipulandum (thumb on top). The fingers were taped to the thumb in order to standardize the grip adopted by the subjects. The manipulandum was mounted on the MR table and was carefully positioned to optimally fit in the scanner and allow free movement in all directions. To provide (continuous) visual feedback on task performance, angular displacement was measured in both planes by two potentiometers (X and Y) integrated in the manipulandum. Visual feedback was provided on a screen using Spike 2 (*Cambridge Electronic Design (CED)*, *Cambridge, UK*) and an analog-to-digital converter board (*Power 1401, (CED)).* On this screen (display dimensions 44×34 cm, screen resolution 1024×768 pixels, Barco, Belgium) both task cue (3×1.5 cm open rectangles) and subject cursor (5×5 mm closed square) positions were projected. Subjects saw the screen via a mirror placed 11 cm from the face. The distance between screen and mirror was 64 cm. If necessary, MR compatible lenses were provided to correct visual acuity of the subject. It was emphasized that subjects should be able to view the full screen.

### Movement Tasks

**Figure 2:**
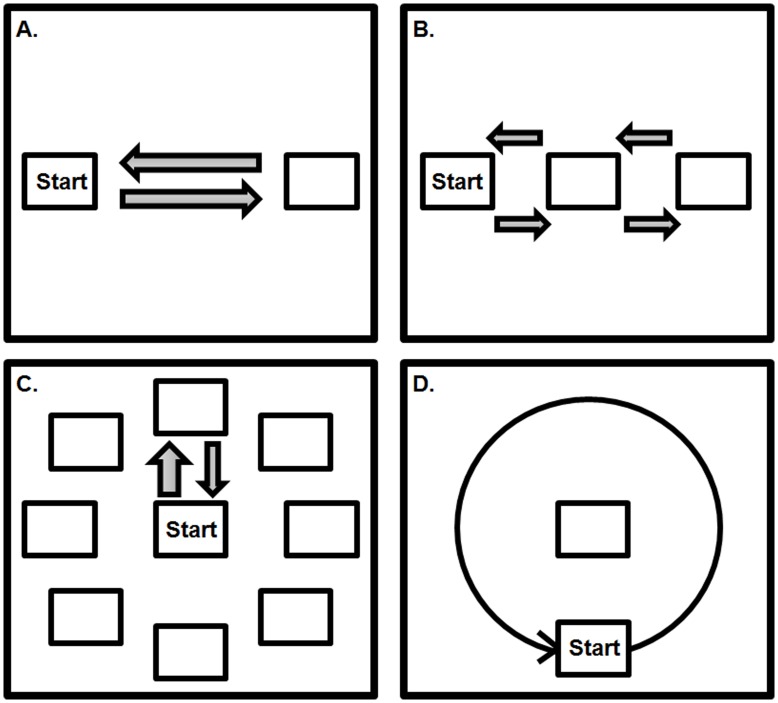
Schematic overview of the four movement tasks. A = ballistic movement consisting of extension and flexion with abrupt movement onset without intentional stops (focused on movement initiation); B = stepwise interrupted movement consisting of flexion-extension with four intermediate stops (focused on movement inhibition); C = centre-out step-tracking, consisting of movement towards one of eight possible directions, stopping at the target location and returning slowly; D = continuous circle movement (wrist circumduction): consisting of following the moving target (clockwise or counter clockwise direction) as smoothly as possible (focused on gradual movement execution).

Subjects performed the movement tasks ballistic movement initiation, stepwise interrupted movement, step-track and continuous circle movement in four runs. Each run encompassed all four movement tasks, each consisting of multiple trials within a block (overview in fig. 2). The four blocks within a run were ordered in fixed-randomized fashion, i.e. the sequence of the four tasks varied for each of the four runs, but was the same for each subject. Blocks of different movement tasks were separated by a 35s rest (see Table 1). Additionally, between each run there was a short break (about 2 minutes) that could be used to communicate with the subjects and give auditory feedback when required. Prior to the start of each run, subjects had to hold their hand in a neutral position, i.e. in the center of the manipulandum, while the center of the screen was adjusted to the position of the cursor corresponding to this neutral hand position by calibration. This was done to ensure anatomic variation of hands did not interfere with task execution. After performing the tasks outside the scanner in sitting position for four runs (these data were analyzed separately [30]), subjects practised the task in a dummy MRI scanner (for at least one run). Just before the scan session there was a short rehearsal of all movement tasks (one run) to ensure subjects remembered task instructions. Task performance was monitored on a computer screen in the MR control room.

**Table 1:** Example task run.

1x Block:							
**1x block ballistic = 5x ballistic (flex-ext)**	+	5x ballistic	+	5x ballistic	+	5x ballistic	rest
**1x block stepwise = 5x stepwise (flex-ext)**	+	5x stepwise	+	5x stepwise	+	5x stepwise	rest
**1x block steptrack = 10x steptracks**	+	10x steptracks	+	10x steptracks	+	10x steptracks	rest
**1x block circles = CCW(10x) - CW(10x) -CCW-CW-CCW**	+	5x 10 circles	+	5x 10 circles	+	5x 10 circles	rest

Example of one task run consisting of one block of each of the four movement tasks. One block of ballistic and stepwise movements consists of 4×5 cycles of flexion-extension and after each 5 cycles there is a short break of 2 seconds (‘+’). One block of steptrack movements consists of 4×10 steptracks, after each 10 steptracks there is a short break (‘+’) of 4 seconds. One block of circle movement consists of alternating cycles (each cycle consists of 10 consecutive circular movements) of counter clockwise (CCW) and clockwise (CW) circles (CCW-CW-CCW-CW-CCW), separated by a short break (‘−’) of 5 seconds. After each block of a movement task there is a rest (‘rest’) period of 35 seconds.

#### Task 1. Ballistic movement initiation (“Ballistic”)

This task involved abrupt initiation of hand movement [9]. First, subjects placed the cursor in the center of the screen (neutral position). Next, a warning cue (a cross at the center of the screen), was presented for 1 second. After disappearance of this warning cue, the initial stimulus directly appeared on the left side of the screen (at 20 degrees from the center of the screen) requiring hand movement from the neutral position towards a flexed position. This flexed position was the starting position for 4×5 consecutive trials of ballistic extension – flexion movement cued by visual stimuli at the right and left side of the screen, respectively (fig. 2A). Subjects were instructed not to intentionally stop on the target but to react to the visual stimuli in an explosive manner and let flexion-extension movements be limited by the (physiological) maximum excursion of the wrist joint. After every 5 trials of extension-flexion there was a 2s break. The inter stimulus interval was 1 second.

#### Task 2. Stepwise-interrupted movement (“Stepwise”)

This task was characterized by abrupt inhibition (stopping) of movement [9]. As for the ballistic task, after presentation of the warning cue subjects had to move their hand from the neutral to a flexed starting position in reaction to an initial flexion target. Again, the flexed position was the starting position for 4×5 consecutive trials of extension-flexion movement cued by visual stimuli. After every 5 trials of extension-flexion (40 seconds) there was a 2s break. In contrast with the ballistic movement, subjects now had to make intentional stops at the extension and flexion targets and additional stops in the center, thereby interrupting the extension-flexion movement abruptly (fig. 2b). The inter stimulus interval was 1 second.

#### Task 3. Step-tracking (“Step-track”)

For the centre-out step-tracking task [33] subjects moved towards the target direction as fast as possible, similar to the ballistic task. The step-tracking task had eight different target directions (corresponding to the cardinal points of a compass), however. The directional component of the step-tracking task, therefore, requires more complex visuomotor integration than tasks 1 and 2. All stimuli had the same distance to the center of the screen (20 degrees). As for the ballistic and stepwise tasks, step-tracking started with the presentation of a warning cue (1 second). One second after disappearance of the warning cue, the target stimulus appeared at one of the eight positions. Subjects were required to move as fast and accurately as possible to the target from the starting position (3×1.5 cm open rectangle in the center of the screen; fig. 2C). Step-tracking requires a larger variety of muscle activity as compared to tasks 1 and 2, since movement in some of the directions requires combinations of, for example, flexion and radial deviation. After moving towards the target, subjects had to hold the cursor in the target box until it disappeared (3 seconds after target appearance) before returning to the center box. Each step-track trial lasted 5 seconds. After every 10 trials of step-tracks, there was a 4s break. One step-track block consisted of 40 trials during which stimuli for the eight different directions were presented in fixed randomized order.

#### Task 4. Continuous circle movement (“Circle”)

The circle task required subjects to perform smooth wrist circumduction movements without intermediate starts and stops, requiring continuous modulation of co-active (synergistic) muscles. This was demonstrated at the behavioral level in a previous study [30]. During this task, subjects had to follow a circling target (at a radius of 20 degrees from the center of the screen) as smoothly and accurately as possible (i.e. subjects had to stay on target). Similar to the step-tracking task, tracking the moving cue in the circle task required directional changes of the wrist. However, in contrast with the step-tracking task, these directional changes are continuous. The starting position of the circle task was located at the lowest point of the circle (fig. 2D). First, the warning cross (in the center of the screen) disappeared; 1 second later the target started moving at constant speed (1 circle/2.9 s), either clockwise (CW) or counter clockwise (CCW). One trial of circle movements consisted of ten full rotations. Each block of the circle task consisted of five alternating trials of CW and CCW circles (CCW-CW-CCW-CW-CCW), separated by 5s breaks.

**Figure 3:**
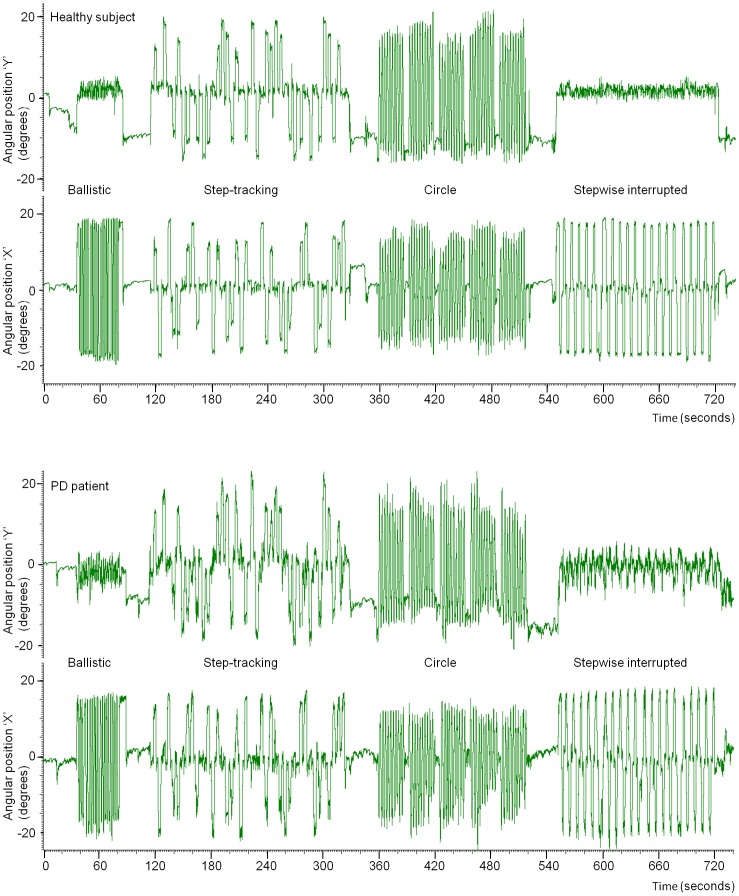
Single subject kinematic data. Typical single subject examples of kinematic data for all four movement tasks during scanning (second run of tasks). X-axis: time (in seconds), Y-axis: the angular position (in degrees) of the hand/wrist during movement execution, derived from the two angular position potentiometers (X and Y) integrated in the manipulandum. Top: healthy subject, bottom: PD patient.

During scanning, subject performance was visually monitored on a second computer in the MR control room. All subjects, both healthy and PD patients responded adequately to all movement cues for each task, i.e. subjects did not miss cues and performed the task according to the instructions. This was confirmed by a global check of the kinematic movement data after the fMRI experiments. Figure 3 illustrates kinematic data of a typical healthy subject and of a PD patient.

### fMRI Data Acquisition

fMRI data acquisition was performed using a 3 Tesla Magnetic Resonance System (Philips, Best, The Netherlands) with a standard 6 channel head coil. T2*-weighted, 3D functional images were obtained using multislice echo planar imaging (EPI) with an echo time (TE) of 30 ms and a repetition time (TR) of 2000 ms. Per TR 39 axial slices (field of view (FOV) 224 mm, flip angle of 5° with a 64×64 matrix and isotropic voxel size of 3.5×3.5×3.5 mm) were acquired. Functional scanning included 370 volumes per block. Additional T1-weighted 3D anatomical scans with an axial orientation and a matrix size of 256×256 mm were obtained to provide anatomical information (isotropic voxel size 1×1×1 mm).

### Data Analysis

Processing of images and statistical analyses were conducted with Statistical Parametric Mapping (SPM) version 5 (2005, Wellcome Department of Cognitive Neurology, London, UK; http://www.fil.ion.ucl.ac.uk/spm). Pre-processing included standard slice time correction, realignment and co-registration of functional and anatomical scans. Images were normalized to the template of the Montreal Neurological Institute (MNI) and smoothed using a Gaussian filter of 8 mm full width at half maximum (FWHM).

We employed a block-design based on the four different movement tasks. The onset of each block was defined by the onset of the go-signal, i.e. the first movement cue for each of the four task blocks within each run. The offset of each block was defined by the end of the last trial in each task block. We modelled the BOLD response by the canonical standard hemodynamic response function (HRF) in SPM5. In SPM5 the block design was convolved with this HRF. It is important to conceive that in this way overall differences between tasks were assessed, not restricted to a specific fraction of time within a task. Statistical parametric maps per subject (first level analysis) were derived using a linear multiple regression model that included movement parameters as regressors of no interest to account for head movement. Comparisons between the four tasks were generated at first level using custom-written scripts (Matlab, Mathworks, Natrick, MA, USA).

To confirm results from our previous study in young healthy subjects [9] and to ascertain that we indeed employed the appropriate movement tasks from which characteristics of movement initiation, inhibition and gradual movement modulation could logically be extracted, we initially made seven comparisons (T-contrasts) between the four conditions in healthy subjects only. To investigate activations related to movement initiation, we employed the comparison (1) ‘Ballistic > Stepwise’. Here, ballistic movement is characterized by abrupt movement initiation (or agonist activity) while stepwise movement particularly includes abrupt stopping achieved by antagonist activity on single-joint level. Although ballistic and circle movements were not balanced for visuomotor demand, (2) ‘Ballistic > Circle’ was assessed to confirm expected activations related to movement initiation. Conversely, the comparison (3) ‘Stepwise > Ballistic’ focused on activation related to movement inhibition. To investigate activations related to gradual movement modulation, we first compared activations related to the two tasks that were characterized by gradual movement adjustment (Circle and Step-track), which additionally required more visuomotor integration, with the two simple movement tasks (Ballistic and Stepwise) (4): ‘Circle + Step-track’ > ‘Ballistic + Stepwise’. As this comparison was expected to include BG activation related to both gradual movement modulation and visuomotor integration, BG activation specifically related to gradually modulated movement was disentangled from visuomotor-related activation by the comparison (5) ‘Circle > Step-track’. To obtain activations specifically related to gradual movement modulation, the comparison (6) ‘Circle > Ballistic’ was made, recognising that this comparison would include additional activations related to stronger visuomotor demand. While similar visuomotor-related activations were expected in the comparisons (6) ‘Circle > Ballistic’ and (7) ‘Step-track > Stepwise’, modulation-related activation was not expected to occur (or less strongly) in the latter. Comparisons 2, 6 and 7 demonstrated that our earlier results in young healthy subjects [9] were confirmed in elder healthy subjects (see Results section: Within group comparisons). We therefore used only four comparisons that were considered crucial to test our hypotheses on differences between groups.

The activation maps of the seven between-task comparisons at first level were entered in separate ANOVAs (flexible factorial design) to statistically compare results within (seven comparisons) and between groups (four out of seven comparisons), at second level. The comparisons of task-related differences between patients and healthy subjects were performed by using exclusive masking (threshold p = 0.05). Note that exclusive masks remove all voxels reaching significance in one contrast that overlap with the significant voxels in the other contrast. In the analyses we focused on the BG/thalamus, premotor cortex (PMC), supplementary motor area (SMA), parietal cortex and cerebellum. To identify activations in cortical areas and the cerebellum, voxel values were thresholded at voxel response height of a liberal p = 0.01 (uncorrected) with an extent threshold of k = 10 voxels. For investigation of activation in the BG and thalamus we used a small volume correction since the BG cover a relatively small region within the brain. This small volume was obtained by using a spherical volume of interest (VOI) with a radius of 30 voxels and a center placed at coordinate [0, 0, 0]; only voxels located within this sphere were analyzed. For the BG and thalamus we used a liberal voxel response height of p = 0.05 (uncorrected and extent threshold of k = 30 voxels). The liberal thresholds were considered valid because we assessed effects in relative small brain regions for which clear hypotheses were formulated [44], [45], particularly concerning movement initiation/inhibition [9], while previous studies indicated that these areas are subject to PD-related changes [46]–[52]. Moreover, given the fact that the general characteristics of the applied movement tasks were highly similar, the small activation differences revealed by the executed comparisons could be more specifically linked to the higher-order task components we looked for. Finally, the various comparisons made within the group of healthy subjects (seven) provided the opportunity to assess consistency in the pattern of activation increases, thus supporting the inference that, although p-values were liberal, these increases represented physiological effects and were not attributed to statistical noise. Activations in other regions were only reported when p<0.001 (uncorrected and extended voxel threshold of k = 10 voxels). Brain regions were identified by rendering group activation maps onto the Automated Anatomical Labeling (AAL) template and Brodmann template in MRICron [53].

## Results

### Subjects

19 healthy subjects and 13 patients with mild to moderate idiopathic PD participated in the study (see table 2 for clinical details, scores are off-medication). One patient was excluded from all analyses because of using anti-Parkinson medication during the experiment. One healthy subject was excluded because of a structural anomaly in the anatomical (T1-weighted) scan. Another patient only finished three out of four blocks; these data were included in the final analysis. Thus, data from 12 patients (age range 38–69, mean: 58.1, SD: 8.8, male (7)) and 18 healthy subjects (age range: 50–69, mean: 58.7, SD: 5.4, male (9)) entered the analysis. Although some of the patients had a long disease duration, clinically, they were in relatively good condition. The clinical characteristics of the youngest patient were similar to those of the older patients and this patient was not known to have genetic mutations. A student t-test revealed that there was no significant difference between the age of healthy subjects and patients (p = 1.00). MMSE scores were comparable between groups; the median MMSE-score was 28 for patients, and 29 for healthy subjects.

**Table 2:** Clinical details of patients with Parkinson’s disease.

Patients	Age	Sex	MMSE	UPDRS (motor)	Laterality (rigidity and bradykinesia scores(UPDRS) upper extremities)	H&Y stage	Disease Duration
PD1	69	M	29	36	left (+2)	3	4
PD2	57	F	29	15	right (+1)	2	11
PD3	48	F	28	18	equal	1.5	7
PD4	60	M	28	12	left (+3)	1.5	4
PD5	60	M	29	18	equal	1.5	11
PD6	64	M	29	23	right (+3)	1.5	4
PD7	69	M	27	26	equal	2	6
PD8	54	M	28	26	left (+1)	1.5	3
PD9	60	F	29	27	right (+2)	1.5	3
PD10	62	F	28	18	right (+1)	2	1
PD11	63	M	28	25	right (+1)	2	11
PD12	38	F	29	14	right (+3)	2.5	3
**Mean**	59	M = 7	28	22	6 right (mean difference = 2);	2,0	6
**SD**	9		1	7	3 left (mean difference = 2); 3 equal	0,5	4

Clinical details of patients with Parkinson’s disease (PD). MMSE = Mini Mental State Examination, UPDRS = Unified Parkinson’s Disease Rating Scale, H&Y = Hoehn and Yahr scale, M = male, F = female. Laterality scores (rigidity and bradykinesia scores of the upper extremities) indicate the difference in scores between right and left side. All scores are off medication.

### Within Group Comparisons: Healthy Subjects

To better understand changed activations in PD, normal task-related activations were first identified in healthy subjects (see also Data analysis).

#### Ballistic movement vs. stepwise interrupted movement

**Figure 4:**
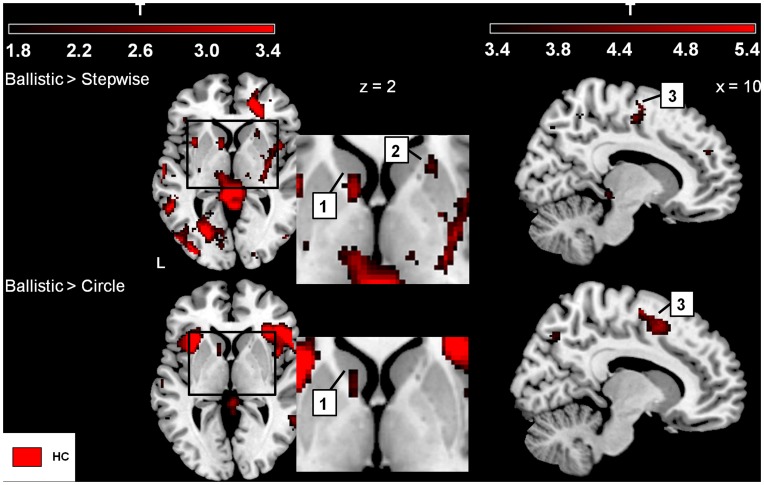
Ballistic initiation versus Stepwise and Circle, respectively (healthy subjects). Increased activations (SPM-T maps) focusing on movement initiation. The color-coded bars at the top of the figure indicate t-map intensities (T = 2.4 corresponds to p = 0.01 (uncorrected)). 1 = caudate nucleus, 2 = (anterior) ventral putamen, 3 = supplementary motor area (SMA). Basal ganglia activation shown at z = 2 mm (the position of the transversal plane relative to the AC-PC plane) with a threshold p = 0.05 (uncorrected and extended voxel threshold of k = 30 voxels), and SMA activation is shown at a threshold p = 0.001 (uncorrected and extended voxel threshold k = 10) shown at x = 10 (position of parasagittal plane relative to the sagittal plane that divides left and right sides of the brain). Left side of the brain is marked ‘L’.

The comparison ‘Ballistic > Stepwise’ (focused on movement initiation) was related to activations in the contralateral (left) substantia nigra (SN), caudate head, bilateral putamen and posterior thalamus. In addition, bilateral cerebellum, (pre-)motor cortex, supplementary motor area (SMA) (BA 6), parietal cortex and contralateral primary sensory (S1) were more activated. Although the comparison ‘Ballistic > Circle’ was not balanced for visuomotor demand, it similarly activated the head of the left caudate and SMA, of which the latter now extended anteriorly into the pre-SMA (fig. 4). Furthermore, ‘Ballistic > Circle’ activated the bilateral anterior thalamus, PMC, cingulate gyrus, bilateral anterior insula and ipsilateral frontal operculum. Conversely, ‘Stepwise > Ballistic’ (focused on movement inhibition), activated the bilateral subthalamic nucleus (STN), ipsilateral pallidum, striatum and dorsolateral prefrontal cortex (DLPFC).

#### Gradual movement modulation and visuomotor integration

**Figure 5:**
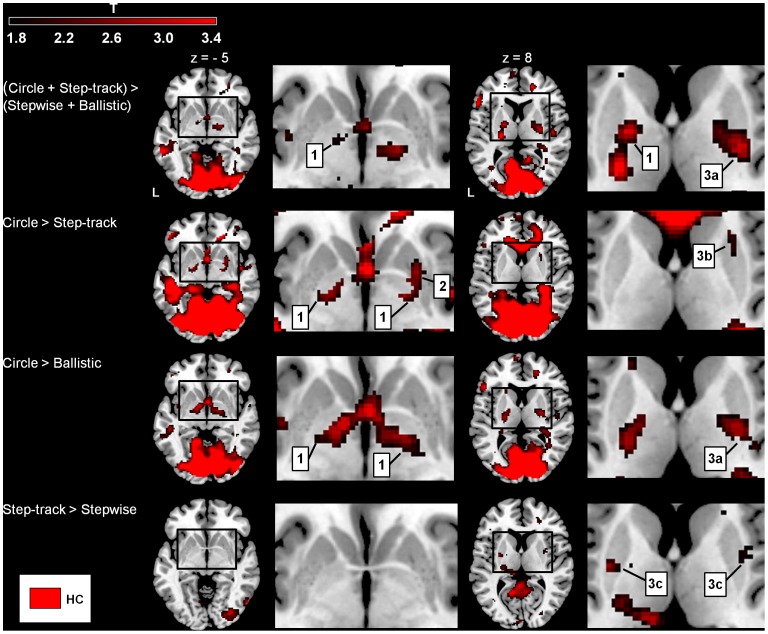
Gradual movement vs. visuomotor control (healthy subjects). Increased basal ganglia (BG) activations (SPM T-maps) for four different comparisons focused on dissociating the role of the BG in organization of gradual movement modulation and visuomotor control. The color-coded bar at the top of the figure indicates t-map intensities (T = 2.4 corresponds to p = 0.01 (uncorrected)). 1 = pallidum, 2 = medial putamen, 3a = dorsal posterior putamen, 3b = dorsal anterior putamen, 3c = dorsal medial putamen. BG activations are shown with a threshold p = 0.05 (uncorrected and extended voxel threshold of k = 30 voxels) at z = −5/+8 (transversal plane relative to the AC-PC plane). Activations were rendered on the standard anatomical template of MRICron [53]. Left side of the brain is marked ‘L’.

The overall comparison ‘Circle+Step-track > Ballistic+Stepwise’ revealed activations related to gradually modulated movement as well as to enhanced visuomotor control. These activations were located in the bilateral pallidum, posterior dorsal putamen, bilateral cerebellum, primary motor cortex (M1), S1 and ipsilateral superior parietal cortex. To investigate which area was specifically related to gradually modulated movement, and not related to differences in visuomotor demand, ‘Circle > Step-track’ was assessed. This comparison yielded activations in the contralateral pallidum and ipsilateral anterior dorsal putamen (fig. 5). Contralateral pallidum activation was also observed in ‘Circle > Ballistic’ and not in ‘Step-track > Stepwise’, thus supporting its specific contribution to gradual movement modulation (fig. 5). ‘Circle > Ballistic’ additionally showed increased activations in the ipsilateral posterior dorsal putamen, (anterior) cerebellum and superior medial frontal cortex, while activations in posterior cortical regions comprised ipsilateral superior parietal cortex and primary visual cortex. These additional activations reflected enhanced visuomotor control. Given the results of the above reported comparisons, activations from the comparison related to ‘Circle > Step-track’ in mid-dorsal putamen, parietal cortex and cerebellum were not unequivocally specific for gradual movement modulation but were also strongly implicated in visuomotor control (fig. 4).

### Between-group Comparisons: PD Patients versus Healthy Subjects

#### Ballistic movement vs. stepwise interrupted movement

**Figure 6:**
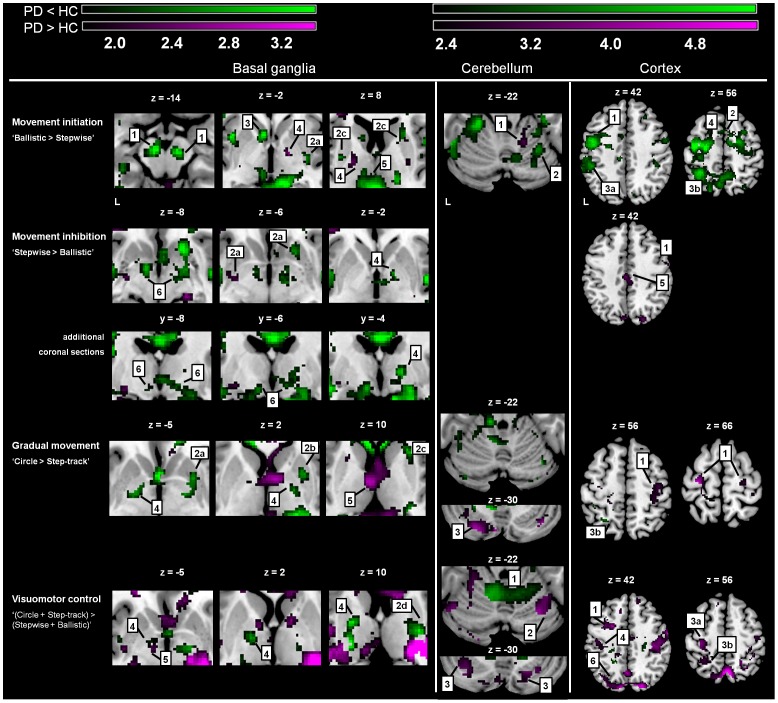
Differences in task-related activations between groups. SPM T-maps of changed activations for three comparisons focused on abrupt movement initiation, movement inhibition and gradual movement modulation. The color-coded bars at the top of the figure indicate t-map intensities (T = 2.4 corresponds to p = 0.01 (uncorrected)). Foci of activation in green: Increased activations in Healthy Controls (HC) that did not occur in Parkinson’s Disease (PD), i.e. PD < HC, purple: PD > HC. Basal ganglia: 1 = substantia nigra, 2a = ventral putamen, 2b = medial putamen, 2c = dorsal anterior putamen, 2d = dorsal posterior putamen, 3 = caudate nucleus, 4 = pallidum, 5 = thalamus, 6 = subthalamic nucleus; Cerebellum: 1 = anterior cerebellum, 2 = posterior cerebellum, 3 = crus; Cortex: 1 = premotor cortex, 2 = supplementary motor area, 3a = inferior parietal cortex, 3b = superior parietal cortex, 4 = primary motor cortex 5 = cingulate gyrus, 6 = parieto-occipital sulcus. Differences in activations between groups were assessed by using exclusive masks (p = 0.05). For activations of the basal ganglia (BG) activations are shown above a threshold level of p = 0.05 (uncorrected and extended voxel threshold of k = 30 voxels), for the cerebellum/cortex activations are shown above threshold level p = 0.01 (uncorrected and extended voxel threshold of k = 10 voxels). The ‘z’ coordinates indicate the position of the transversal planes relative to the AC-PC plane. Activations were rendered on the standard anatomical (ch2) template of MRICron [53]. Left side of the brain is marked ‘L’.

For ‘Ballistic > Stepwise’ (focused on movement initiation) patients had decreased BG activations, distributed over SN, striatum and posterior thalamus (exclusive masking p = 0.05, fig. 6). Decreased activation was also seen in lateral premotor areas, SMA (BA6), parietal cortex (BA7) and cerebellum. Increased activations were found in the pallidum bilaterally (fig. 6). For ‘Stepwise > Ballistic’ (focused on movement inhibition), patients had decreased activations of bilateral STN, ipsilateral pallidum, and bilateral insula (fig. 6), whereas activations of the contralateral putamen and ipsilateral caudate were increased. Additionally, patients showed increased activation of the cingulate gyrus and ipsilateral (pre-) motor cortex (overview in tables 3–4).

**Table 3:** Basal ganglia and cerebellar activations: Healthy subjects vs. Patients with Parkinson’s disease.

Basal ganglia	Ballistic > Stepwise		Stepwise > Ballistic		Circle > Step-tracking		Stept + Circ > Ball+ Stepw	
*contralateral (left)*	HC (PD↓,PD↑)	X,Y,Z	HC (PD↓,PD↑)	X,Y,Z	HC (PD↓,PD↑)	X,Y,Z	HC (PD↓,PD↑)	X,Y,Z
ventral caudate nucleus	+ PD**↓**	−6,12, −2						
ventral putamen			PD**↑**	−24, −8, −6				
dorsal putamen	+ * PD**↓**	−24,−2, 16			PD**↑**	−24, 6, 16	+ ** PD**↓**	−20, −4, 10
pallidum	PD**↑**	−20, −10, 10			+ PD**↓**	X	+ PD**↓**	−20, −4, 8
subthalamic nucleus			+ PD**↓**	−8,0,−12				
substantia nigra	+ PD**↓**	−8, −14, −14						
ventral ant. thalamus					PD**↑**	−4, −8, 10		
dorsal thalamus (pulvinar)	+ PD**↓**	−2, −16, 4	PD**↑**	−18, −26, 12			PD**↑**	x
*ipsilateral (right)*								
ventral caudate nucleus			+ PD**↓**	12, 24, 4				
ventral putamen			+ PD**↓**	−22,16,8				
dorsal caudate nucleus			PD**↑**	14, 6, 12				
medial putamen	PD**↓**	26, −8, 4			+ PD**↓**	26, 6, −2		
dorsal putamen	+ * PD**↓**	24, 16, 6			+ * PD**↓**	26, 16, 16	+ ** PD**↓**	26, −10, 10
pallidum	PD**↑**	16, −4, 0	+ PD**↓**	16,−2,2	+ PD**↓**	20,−6,2	+ PD**↓**	20, −4, 8
subthalamic nucleus			+ PD**↓**	−18, 0, −10				
substantia nigra	+ PD**↓**	8, −14, −14						
ventral ant. thalamus								
dorsal thalamus (pulvinar)	+ PD**↓**	18, −20, 10						
**Cerebellum**								
*contralateral (left)*								
lobus anterior	+ PD** = **				+ PD** = **		+ PD**↓**	−10, −52, −22
lobus posterior	+ PD**↓**	−12, −62, −38			+ PD** = **		+ PD**↑**	−42, −66, −18
crus					+ PD**↑**	−24, −78, −28	PD**↑**	−20, −86, −22
*ipsilateral (right)*								
lobus anterior	+ PD**↑**	10, −64, −20			+ PD** = **		+ PD**↓**	24, −48, −24
lobus posterior	+ PD**↓**	22, −68, −22			+ PD** = **		+ PD**↑**	20, −72, −26
crus					+ PD**↑**	38, −72, −30	+ PD**↑**	28, −70, −30

Overview of activation in the basal ganglia (BG) and cerebellum for four contrasts and differences between healthy subjects (HC) and patients with Parkinson’s disease (PD) concerning abrupt movement initiation and inhibition (BG: p<0.05 (extended threshold: k = 30 voxels), cerebellum: p<0.01, (extended threshold: k = 10 voxels)). ‘Ball’ = ballistic movement, ‘Stepw’ = stepwise movement, ‘Stept’ = step-tracking movement and ‘Cir’ = circle movement. ‘+’ = area activated in HC. In case of a significant difference in region-specific activation between patients and healthy subjects, this is indicated using arrows: **↓** =  PD< HC, **↑** =  PD> HC.

* = anterior dorsal putamen,

** = posterior dorsal putamen. ‘x’ indicates those BG regions that were part of a larger cluster and for which a specific coordinate could not be found.

**Table 4:** Cortical activations: Healthy subjects vs. Patients with Parkinson’s disease.

Cortex	Ballistic > Stepwise		Stepwise > Ballistic		Circe > Step-tracking		Step-tracking + Circle > Ballistic + Stepwise	
	HC (PD↓,PD↑)	X,Y,Z	HC (PD↓,PD↑)	X,Y,Z	HC (PD↓,PD↑)	X,Y,Z	HC (PD↓,PD↑)	X,Y,Z
Cingulate gyrus			+ PD**↑**	2, −30, 32			PD**↑**	−2, −28, 36
SMA	+ PD**↓**	0, 4, 62						
prefrontal							+ PD**↑**	−2, 66, 12
*contralateral (left)*								
Operculum (anterior)	+ PD**↓**	−48, 22, 30						
Operculum (posterior)								
Insula			+ PD**↓**	−40, −34, 20			PD**↑**	−38, −8, 8
DLPFC								
PMC	+ PD**↓**	−38, −10, 54	PD**↑**	−34, −4, 40	PD**↑**	−30, −20, 64		
primary motor ctx	+ PD**↓**	−50, −8, 40					+ PD**↑**	−26, −28, 50
primary sensory ctx	+ PD**↓**	−52, −22, 30			+ PD**↑**	−40, −16, 42	+ PD**↑**	−38, −28, 50
inferior parietal ctx	+ PD**↓**	−28, −52, 54						
superior parietal ctx					+ PD**↓**	−30, −56, 62	PD**↑**	−22, −50, 58
Parieto-occipital sulcus			PD**↑**	−4, −80, 40			PD**↑**	−8, −82, 40
Occipital V1/V2	+ PD**↓**	−46, −80, −2			+ PD**↓**	−32, −82, 0	+ PD =	
*ipsilateral (right)*								
Operculum (anterior)	+ PD**↓**	42, 10, 26						
Operculum (posterior)			+ PD**↓**	50, −12, 10				
Insula			+ PD**↓**	48, −6, 0			PD**↑**	32, −20, 12
DLPFC			+ PD**↓**	30, 20, 34				
PMC	+ PD**↓**	40, 6, 34	PD**↑**	24, −30, 74	PD**↑**	24, −22, 66	PD**↑**	
primary motor ctx			PD**↑**	60, −2, 38	PD**↑**	54, −12, 38	PD**↑**	44, −20, 44
primary sensory ctx	+ PD** = **				PD**↑**	38, −34, 62	+ PD**↑**	38, −18, 40
inferior parietal ctx	+ PD**↓**	42, −40, 50						
superior parietal ctx					+ PD**↓**	−24, −58, 54	+ PD**↑**	34, −44, 62
Parieto-occipital sulcus			PD**↑**	10, −66, 58			PD**↑**	16, −80, 34
Occipital V1/V2					+ PD**↓**	38, −78, −4	+ PD =	

Overview of activation in the cortex for four contrasts and differences between healthy subjects (HC) and patients with Parkinson’s disease (PD) concerning abrupt movement initiation and inhibition (supplementary motor area (SMA), premotor cortex (PMC) and parietal cortex <0.01, other regions p<0.001 (extended threshold: k = 10 voxels)). DLPFC = dorsolateral prefrontal cortex. ‘+’ = area activated in HC. In case of a significant difference in region-specific activation between patients and healthy subjects, this is indicated using arrows: **↓** =  PD< HC, **↑** =  PD> HC.

#### Gradual movement modulation and visuomotor integration

The comparison ‘Circle+Step-track > Ballistic+Stepwise’, focusing on both gradual movement modulation and visuomotor control, revealed decreased activation of the bilateral pallidum and dorsal putamen in patients (fig. 6). Patients had increased activations in the contralateral thalamus and widely distributed in the cortex, including the sensorimotor cortex. For this comparison, patients showed decreased activations in the anterior lobulus of the cerebellum, while increases were seen in the posterior cerebellum (fig. 6). For gradually modulated movement (‘Circle > Step-track’), patients showed decreased activation in the pallidum bilaterally, ipsilateral in the (right) mid- and anterior dorsal putamen and (superior) parietal cortex (BA7). The decreased anterior cerebellar activation associated with visuomotor control, was not seen in ‘Circle > Step-track’ in patients. Similar to the visuomotor-associated activation increase, increased activation in ‘Circle > Step-track’ included the posterior cerebellum. Subtle activation increases were additionally seen in the contralateral anterior putamen, (anterior) thalamus, while bilateral increases in the (pre-) motor cortex were more prominently present (fig. 6 and tables 3–4).

## Discussion

The four different movement tasks employed in the present fMRI study were all executed at single joint level (the wrist). This similarity between tasks enabled distinction of functional segregation within the BG underlying three different modes of motor action as well as enhanced visuomotor control. In PD, both decreases and increases of task-related BG and associated cortical activations were seen relative to activations in healthy subjects. Normal movement initiation was characterized by antero-ventral striatum and SN activations; movement inhibition was dominated by activation of STN and pallidum, in line with the results of our previous findings in healthy subjects [9]. Gradually modulated movement was related to activation of the pallidum and antero-dorsal putamen. This anterior putamen activation was located in the ipsilateral (right) hemisphere while right postero-dorsal putamen activation was associated with a stronger demand on visuomotor integration. Comparison of these healthy subject activations with the task-induced effects in patients revealed that patients had (i) reduced striato-cortical and SN activations together with increased pallidum activation for movement initiation, (ii) decreased STN activation for movement inhibition and (iii) decreased pallidum activation for both inhibition and gradually modulated movement. In contrast to the distinct cortical decreases in PD movement initiation, both cortical decreases and increases were seen when patients performed the tasks characterized by inhibition and gradual modulation. Regarding the tasks with enhanced visuomotor demand, cortical activations were increased in PD compared to healthy subjects.

### Initiation, Inhibition and Gradual Movement Modulation

In healthy subjects, abrupt movement initiation was characterized by activation of the antero-ventral striatum (caudate head) and SN, without pallidum activation, while the latter was clearly present in movement inhibition and gradual modulation. Striatal activation without a specific pallidum contribution fits the concept that initiation in ballistic movement is particularly characterized by the first agonist burst of a triphasic pattern [54]. The amplitude of this initial burst has been proposed to reflect a measure of the degree to which muscle force is scaled to achieve the movement prepared for [54], [55]. The initial stage of agonist contraction is associated with general antagonist relaxation. This may imply that for abrupt movement initiation the pallidum is not recruited for fine-tuned partial inhibition normally enabling precise movement selection [13], [56]–[59]. The co-occurence of activations in the medial segment of the anterior striatum (caudate) and (pre)SMA in healthy subjects in the present study may further underscore a common contribution to movement initiation [60], [61]. In addition to medial frontal-striatal activations SN involvement in specifically movement initiation was previously described [62]. The association of caudate and SN activations with movement onset may reflect the start of a neuronal timing process [63], [64]. For ballistic movement, one may speculate that such timing concerns e.g. the estimated duration of agonist contraction.

When treating activations in a small region as the STN there is a potential methodological pitfall caused by possible misregistration and smoothing. Nevertheless, activation of putative STN and pallidum during a task with repeated movement inhibition is consistent with the hypothesis that these areas play an important role in selection of appropriate movement by inhibiting unwanted movement. In other words, these areas function as a a ‘braking’ system [13], [56]–[59], [65]. It should be conceived that full inhibition in our paradigm concerned stopping of ongoing movement. In contrast to the other three tasks, no increases of striatal activations were seen in this condition, suggesting a ‘bypass’ using direct cortico-STN connections [24], [66], [67]. Indeed, in a ‘hyperdirect pathway’, cortical information is directly transmitted via the STN to the internal pallidum and SN [67]. Bilateral STN activations related to inhibition might be explained by its ‘stop-all’ function [68]: the bilateral STN receives direct (bilateral) cortical input via the hyperdirect pathway and, during movement inhibition, inhibits the brain areas normally involved in motor tasks. In stepwise movement, inhibition implies full stops without maintained specification of selective movements. Here, activation of the lateral prefrontal cortex related to full movement inhibition, is in accordance with other studies on movement inhibition [5], [18], [69], [70]. From the characteristics of abrupt movement changes in the stepwise task one might infer that this task does not require an elaborate routing within the striatum for gating cortical information into direct and indirect basal ganglia pathways [24], [71], [72], because such striatal gating is particularly expected in association with the modulatory role of the pallidum in movement selection based on partial inhibition [73]. The latter is the case in the circle task, which indeed recruited BG activations in pallidum and (dorsal) anterior striatum, without activations in either STN or SN.

### Visuomotor Control

In healthy subjects a functional association was found between the postero-dorsal striatum (particularly right-sided) and visuomotor integration. Ipsilateral activation is concordant with involvement of right-sided cortical areas in visual processing and spatial attention. Indeed, increased visual activations were demonstrated in the more complex visuomotor tasks (fig. 5). Additionally, these findings are consistent with visual and parietal cortical regions generally having strong input to posterior striatum segments [74]–[76]. Moreover, cortical regions that are heavily interconnected project to common targets in the striatum [14], [34], [77], thereby placing the BG in a central position for facilitation and regulation of cortico-cortical interactions [14], [78], [79]. Depending on the BG - cortical loops involved, such interactions may, thus, be implicated in both internally-guided and visually-guided movements [14]. In our study, additional cerebellar involvement in the more complex visuomotor control conditions emphasizes its role in motor control by (feedforward) processing of sensorimotor information [80]–[82]. The regulation character of cerebellar functions, both in feedback and feedforward modes, supports the cerebellum being particularly engaged in the performance of externally-guided movement control [14], [83]–[85].

### Changes of Activation in PD

In general, healthy subject activations in BG key structures related to respectively movement initiation, inhibition, gradual movement modulation and visuomotor integration were reduced in patients. Reduced activation at various task-specific locations within the striatum is consistent with the classical feature of impaired striatal function in PD. In existing models of PD, such striatal dysfunction induces disinhibition within the BG associated with a disbalance between direct and indirect pathways, resulting in an enhanced inhibitory BG outflow to successively the thalamus and cortex [11], [22], [24], [31], [32], [62], [86]. In the present study, we indeed observed increased pallidal activations in PD during movement initiation, which is consistent with these models. However, patients also had *reduced* pallidum and STN activations during movement inhibition. Although the latter might reflect the increased vulnerability to fail at stopping ongoing movements [87]–[90], the ‘classic’ model predicts increased STN activation in PD. However, it should be kept in mind that this model describes a static condition while our findings were obtained in the dynamic circumstances of task performance. For example, direct cortical effects on the STN may strongly vary depending on the actual state of cortical activations [15], [67], [91], [92].

This putative larger variation in cortical influences on BG activation in PD, compared to healthy subjects, is illustrated by the different profiles of cortical changes seen in the different tasks. In movement initiation, general cortical decreases were seen in PD, most obviously in lateral and medial premotor regions as well as various (contralateral) parietal regions. In the classic model, this is well explained by reduced BG- thalamic outflow. Moreover, these decreases were consistent with previously described cortical metabolic changes in PD resting-state conditions [93]–[95]. The other tasks additionally showed cortical increases in PD relative to healthy subjects, which was particularly evident in lateral premotor and posterior parietal regions during stronger demands on visuomotor integration. Such increases might fit the enhanced responsiveness to external stimuli in PD [35], [36], [96]. In this respect, reduced inhibitory BG outflow to the thalamus might lead to non-specific cortical facilitation with almost reflex-like cortico-cortical interactions, which complicates performance of e.g. tasks with incongruent visual and motor parameters [34], [36], [97], [98]. Moreover, increased cortical activation may generate an increased excitatory load onto the BG. Elucidating the temporal dynamics in cortico-BG activations, therefore, is a challenge for future fMRI research addressing the pathophysiological mechanisms underlying PD. It should be realized, in this respect, that our results concern the distribution of regional activations, detected by (changes in magnitude of) local BOLD responses. In addition to this classic method, temporal synchronization in spatially distributed BOLD fluctuations may further reveal subtle interactions within functional networks [99].

To what extent posterior cerebellar activation increases in PD visuomotor control are either a direct consequence of increased cortical activation or a compensational mechanism to altered BG functioning [50] cannot be unequivocally concluded. These cerebellar increases during the more complex movement tasks may be due to increased reliance on visuospatial processing, which may be facilitated by reciprocal connections between the cerebellum and the BG input nuclei (as known from animal studies) [100]. Aside from the impact of increased complexity, a theoretical consequence of impaired movement planning would be more corrective adjustments, in which increased cerebellum activation might reflect a compensational strategy for PD-related striatal dysfunction [50].

A more general discussion point, applicable to all studies investigating changes in movement-related activation patterns in PD patients, is whether these changes are related to differences in task-execution or to disease-induced changes in the cerebral organization of movement. This is hard to distinguish, because changes in movement performance at the behavioral level are an integral part of the *movement* disorder. On the other hand, in the present study kinematic data demonstrated that in general tasks were performed as requested by both healthy subjects and patients (fig. 3). Furthermore, it is well-accepted that the BG are important in movement organization and, moreover, that PD patients have specific disease-related changes in BG function. Thus, our findings of differences in activations in the BG and interconnected circuitry are likely to be related to the disease-related changes in movement organization.

### Conclusion

In the present study we disentangled cerebral activation patterns related to various conditions of movement selection at single-joint level, varying from abrupt initiation and inhibition to gradual modulation of movement. Compared to healthy subjects, PD patients showed region-specific changes in activation during all three types of movement indicating that impaired movement organization in PD can not be attributed exclusively to increased inhibitory output of the BG. Instead, our findings appear to be better explained in the context of changed dynamic interactions between excitation and inhibition within circuitries comprising both BG and cerebral cortex. An important message of the present study is that not only decreases, but also task-dependent increases in cortical activation may occur in PD as compared to healthy subjects. Such task-specific dynamics emphasize the need to consider the effects of hyper-fluctuating cortical inputs to striatum and STN in particular. We therefore propose that, in addition to the current static model of direct and indirect BG pathways, a dynamic model would better link the expression of symptoms with altered neuronal network functioning in the Parkinsonian state.
